# Factors affecting the uptake of new medicines: a systematic literature review

**DOI:** 10.1186/1472-6963-14-469

**Published:** 2014-10-20

**Authors:** Ágnes Lublóy

**Affiliations:** Department of Finance, Institute of Finance and Accounting, Corvinus University of Budapest, Fővám tér 8, Budapest, 1093 Hungary

**Keywords:** Diffusion, Drug characteristics, Early adopters, New drugs, Patient characteristics, Pharmaceutical marketing, Practice characteristics, Prescriber characteristics, Social networks of doctors, Systematic literature review

## Abstract

**Background:**

The successful diffusion of new drugs is crucial for both pharmaceutical companies and patients—and of wider stakeholder concern, including for the funding of healthcare provision. Micro-level characteristics (the socio-demographic and professional characteristics of medical professionals), meso-level characteristics (the prescribing characteristics of doctors, the marketing efforts of pharmaceutical companies, interpersonal communication among doctors, drug attributes, and the characteristics of patients), and macro-level characteristics (government policies) all influence the diffusion of new drugs. This systematic literature review examines the micro- and meso-level characteristics of early prescribers of newly introduced drugs. Understanding the characteristics of early adopters may help to speed up the diffusion process, promote cost-efficient prescribing habits, forecast utilisation, and develop targeted intervention strategies.

**Methods:**

The PubMed and Scopus electronic databases were chosen for their extensive coverage of the pertinent literature and used to identify 205 potentially relevant studies by means of a four-layered search string. The 35 studies deemed eligible were then synthetized carefully and critically, to extract variables relevant to this review.

**Results:**

Early adoption of new drugs is not a personal trait, independent of drug type, but early adopters share both micro- and meso-level characteristics. At prescriber level, doctors’ interest in particular therapeutic areas, participation in clinical trials, and volume of prescribing—either in total or within the therapeutic class of the new drug—increase the likelihood of early adoption. The marketing efforts of pharmaceutical companies and doctors’ professional and social interactions leading to prescribing contagion are very powerful predictors of new drug uptake. At patient level, doctors with younger patients, patients with higher socioeconomic statuses and/or patients with poorer health statuses are more inclined to prescribe new drugs early. In contrast, the socio-demographic characteristics of prescribers and many practice-related factors play little role in the adoption process.

**Conclusions:**

The most powerful predictors of new drug uptake include the doctors’ strong scientific commitment, high prescribing volume in total or in within the therapeutic class of the new drug, high exposure to marketing, and intense communication with colleagues.

## Background

In most industrialised countries, drug expenditure as a percentage of overall healthcare cost is increasing rapidly. Changing demographics—ageing populations with increased morbidities—and a rise in the number of drugs per patient contribute obviously to growing prescription costs. However, the key factors in rising drug expenditures are the greater variety and availability of new, expensive drugs and the higher relative costs of pharmaceuticals [1, 2].

Innovation and the successful diffusion of new drugs are critical for the financial performance of pharmaceutical companies—as well as the health of patients. New drug diffusion is determined mostly by the strategies of pharmaceutical companies, government policies, as well as the behaviour of medical professionals. This article concentrates on the last, through a systematic review of the literature using behavioural (prescribing) data.

Although doctors consider each new drug on its individual merits, some seem more predisposed to adopt new drugs than others. This article sets to analyse whether early prescribers of newly marketed drugs are consistently associated with potential prescriber, practice, and patient characteristics—as well as with new drug attributes. To the author’s knowledge, this article is a pioneering attempt at documenting the early adopter characteristics identified by empirical research, thus bringing a valuable contribution to the literature on new drug diffusion. To this end, it synthetizes findings across various countries, drug types, and prescribers—it does not analyse specific drugs or certain specialist groups in isolation.

At micro level, this article sets to analyse the socio-demographic and professional characteristics of early prescribers of newly marketed drugs—as compared to majority and late prescribers. These micro-level characteristics may be defined as characteristics related to the doctors’ personality. At meso level, this article aims to consider quantitatively measurable patient, practice, and drug characteristics and analyse the roles marketing and interpersonal communication play in the adoption process. These meso-level characteristics describe the working environment in which the doctors practice.

At macro level, government agencies and health care organizations are also major influencers as they set the regulatory environment with which pharmaceutical companies and doctors have to cope. While acknowledging the crucial role of macro-level factors in making new medicines available, this article does not aim to review their influence on new drug diffusion.

In this review the terms ‘new drug’ and ‘newly marketed drug’ are used as synonyms and refer to a drug that has been recently approved and introduced to the market. New drugs might be highly innovative drugs—first-in-class drugs with new ATC (Anatomical Therapeutic Chemical Classification System) codes. New drugs might also be me-too or follow-on drugs, which enter the market in an already existing drug class and are chemically very similar to already approved drugs. In this review only highly innovative drugs are considered as pharmaceutical innovations.

Knowing which factors determine new drug uptake is important for several reasons.

First, it might help *speeding up diffusion*. Although companies come up each year with several new drugs [3], their implementation is often delayed [4]. Where new drugs expand therapeutics in areas of yet unmet clinical need, accelerated adoption benefits both medicine and society. If budget allows, new drugs should be offered fast and homogeneously to the population in need—a strategy built upon the key determinants of new drug diffusion is required.

Second, it might help *promoting cost-efficiency*. Healthcare systems worldwide operate with limited financial resources. If new newly marketed drugs are cost efficient —the health status of patients is either maintained at a lower cost, or a better health status is achieved from the same budget— policy makers should encourage the adoption of these cost efficient new drugs to assure the widest accessibility to the particular therapy from the limited healthcare budget [5]. The identification of key determinants might support policy makers in this effort.

Third, it might help *forecasting utilisation*. Accurate prediction is important not only for pharmaceutical companies, but also for healthcare professionals and policy makers in charge of healthcare budget planning.

Fourth, it might help *developing targeted detailing and continuing medical education (CME)*. Where the adoption of new prescription drugs varies among doctors, there is significant potential for targeted pharmaceutical company intervention and CME. Distinguishing between doctors who prescribe new drugs early and those who prescribe them late or never enables targeted intervention through relevant, tailored information—as well as economies of both time and money. In general, detailing and CME should promote appropriate use of new drugs, through prescription of the most efficient/least expensive available alternatives [6].

This review is structured into five sections. Following this ‘Background’, the second section (‘Methods’) presents the research strategy adopted to identify relevant literature. The third section (‘Results’) describes the characteristics of the eligible studies, and summarises the research evidence. At prescriber level, this section discusses characteristics of early adopters with respect to socio-demography, scientific orientation, prescribing pattern, exposure to marketing, and social interaction. The influence of certain practice characteristics, drug attributes, and patient-related factors on the adoption of newly marketed drugs is also discussed in this section. The fourth section (‘Discussion’) elaborates on the variables that produce consistent prediction of new drug uptake and suggests directions for future research. The fifth section (‘Conclusions’) summarises the research findings.

## Methods

### Search strategy and literature selection

This review focuses on literature assessing the prescription of new medicines in either primary or secondary care, with time, geography, and language of no specific interest. The databases used were PubMed and Scopus, and the search was conducted on 24 July 2013. All articles added to the databases until this date were included. PubMed contains journal citations and abstracts for biomedical literature from around the world, whereas Scopus is the largest all-science database of peer-reviewed research literature. The final search string was determined after an initial broad search of the literature using the keywords summarised by category in Table 1. Studies were retrieved if either abstract or main text included at least one keyword from each of the four major categories. Their bibliographies—as well as relevant literature reviews—were rigorously checked to identify further studies. Citations of these studies were also screened, through Google Scholar, with the same aim.

**Table 1:** **Summary of keywords for the search strategy**

Category	Keywords
Object (abstract)	new ATC, new drug(s), new medicine(s), new medication, new substance(s)
Process (abstract)	adopt(ed), adoption, diffuse, diffusion, uptake
Actor (abstract)	doctor(s), general practitioner(s), GP(s), physician(s), specialist(s), SP(s)
Data and method (text)	claim(s), county, logit, nation, odds, population, prescribing, prescription(s), quantitative(ly), region, registry, regression, survival

In alphabetical order, by keyword. The search strings are available from author on request.

Studies were downloaded and screened for eligibility along four criteria:

1. The study was published in a peer-reviewed journal.
2. The study evaluated factors affecting the uptake of new medicines.
3. The study used prescription data from national registries, pharmacy offices, or administrative claims databases. Prescription data reflect the realities of prescribing decisions—including the influences associated with marketing activities (sales representatives, advertisements, and patient requests), evidence bases (peer-reviewed journals and scientific meetings), peer pressures, and regulatory environments. Prescription data also reflect patient characteristics as well as the personal and behavioural traits of prescribing doctors. Qualitative study designs (interviews, focus groups, surveys, and mail questionnaires) were not eligible for inclusion, since methodological drawbacks call their findings into question. A retrospective study based on self-report is at risk of recall and social desirability biases—rather than what actually occurs in practice, surveys and interviews may simply capture normative responses and expressed attitudes [7–9]. Decision making may involve subconscious factors or factors which prescribers—for whatever reasons—choose not to disclose [10]. To avoid screening numerous studies designed qualitatively, methodology-related keywords were added to the search strategy. Some studies complemented prescription data with survey data [7, 11–15]—nevertheless, since the act and timing of adoption are free of recall bias, they were deemed eligible for review.
4. The study concerned at least one significant prescriber or practice characteristic. Studies solely concerning patient characteristics—such as age, gender, and comorbidity—or drug characteristics—such as safety and efficacy—were excluded from this review.

### Data extraction

An Excel database was created to chart the characteristics of the eligible studies and the variables relevant to this review—it included information on the journal where the study was published, study population, country where the study was conducted, analytical method, and the variables the study assessed for impact on new drug uptake. The charted variables were grouped into four categories—prescriber, practice, patient, and drug characteristics. Standard database functions were used for analysis.

## Results

### Search flow

**Figure 1:**
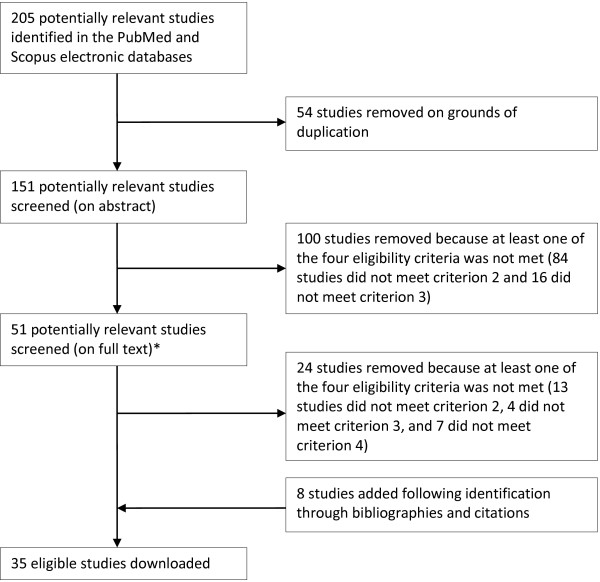
**Flow diagram of the search strategy.** *The full texts of the 51 potentially relevant studies were assessed by Ágnes Lublóy (100%) and Gábor Benedek (12%). The colleagues agreed on all studies reviewed together.

As Figure 1 shows, 178 studies were removed from the initial 205—54 on grounds of duplication and 124 because at least one of the four eligibility criteria was not met—and 8 were added, following identification through bibliographies and citations. Table 2 summarises the remaining 35 eligible studies [1, 5–7, 11–41] in terms of location and size of the sample population, type and number of study drugs, methodology, and factors that might influence new drug uptake.

**Table 2:** **Key characteristics of the eligible studies**

Authors	Population	Drugs	Methods	Variables
Álvárez and Hernández 2005 [16]	32 healthcare centres, 313321 inhabitants, Spain	50 new drugs	multiple linear regressions	*Practice characteristics*: size (number of doctors and number of patients), location (urban or rural), number of years functioning as a primary care centre, *pharmaceutical expense per secured patients*, *number of consultations per doctor*
				*Patient characteristics*: *proportion of patients in pension*
Behan et al. 2005 [17]	126991 inhabitants, 134 full-time equivalent GPs, Australia	2 new drugs (celecoxib and rofecoxib)	comparison of means (Student’s t-test)	*Practice characteristics*: location (urban or rural)
Bourke and Roper 2012 [18]	616 GPs and all their prescriptions, Ireland	6 new drugs, from 6 therapeutic classes	survival analysis	*Prescriber characteristics: age*, *portfolio width*, savings made from meeting prescribing targets, *GP being an early adopter in at least one of the other five study drugs*
				*Practice characteristics*: employee composition* *(practice with nurse, practice with secretary)*, practice with in-house dispensary, location (urban or rural)
				*Drug/market characteristics*: *time-variant percentage of GPs who have adopted the study drug*
Coleman et al. 1966 [7]	125 GPs (prescriptions and interviews) and 103 SPs (interviews), four small cities in Illinois, US	1 new drug (tetracycline, a broad-spectrum antibiotic)	elementary statistics	*Prescriber characteristics*: *age*, *prescribing volume in the therapeutic class of the new drug*, *speciality*, number of contacts with drug representatives, *number of professional journals read*, number of pharmaceutical house organs read, *number of speciality meetings attended*, number of non-speciality meetings attended, *number of hospital meetings attended*, number of county medical society meetings attended, *perceived scientific orientation*, *social position in advisor, discussion, and friendship networks*
				*Practice characteristics*: *type (solo or group/partnership)*
				Note: The survey questionnaire resulted in a very large number of variables—only those most frequently discussed in the relevant literature are reported here
Corrigan and Glass 2005 [19]	4216 doctors, US	38 new compounds	analysis of covariance (ANCOVA) model	*Prescriber characteristics*: gender, age, *board certification*, *hospital affiliation*, *type of doctor (trialist or control)*, *prescribing volume*
Dybdahl et al. 2004 [20]	191 practices, 470000 inhabitants, Denmark	14 new drugs, grouped in 4 categories	Pearson’s correlation coefficient	*Practice characteristics*: type (solo or group/partnership)
				*Drug characteristics*: prescription cost*
Dybdahl et al. 2005 [21]	191 practices, 470000 inhabitants, Denmark	14 new drugs, grouped in 4 categories	multiple linear regressions	*Practice characteristics*: prescribing volume within the therapeutic class of the new drug, number of patients eligible for the new drug, prescribing volume of all other drugs, number of all other patients
Dybdahl et al. 2011 [22]	68 GPs, Denmark	2 new drug groups (COX-2 and AT-II)	univariate and multivariate linear regressions	*Prescriber characteristics*: perceived scientific orientation, perceived need for continuing medical education (CME), current CME activities*, previous hospital employment
Florentinus el al 2007 [23]	86 GPs, 13997 patients, the Netherlands	5 new drugs, from 5 therapeutic classes	logistic multilevel model	*Prescriber characteristics*: *quality of pharmacotherapy audit meetings (PTAMs)*, PTAM composition (number of pharmacists and pharmacies, *total number of participants*, *number of GPs*)
García et al. 2000 [24]	74 GPs and SPs (paediatrics), Spain	28 new drugs, 10 with therapeutic novelty and 18 without	univariate and multivariate linear regressions	*Prescriber characteristics*: age, gender, speciality, type of contract (permanent or temporary), *number of workplaces*, *drug expenditure captured by the deviation from the district’s mean*
				*Practice characteristics*: *management (reformed or non-reformed)*, region
Garjón et al. 2012 [25]	1248 doctors, Spain	8 new drugs, suitable for both primary and secondary care	survival analysis	*Prescriber characteristics*: *speciality*
				*Drug characteristics*: *therapeutic novelty*
Glass 2003 [26]	1876 doctors, US	new drugs for the outpatient treatment of 8 disorders or diseases	comparison of means (Fischer’s least significant difference method)	*Prescriber characteristics*: *investigator type (phase IIIb investigator, phase IV investigator, non-investigator)*
Glass 2004 [27]	2108 clinical trial investigators, US	72 new compounds	multiple linear regressions	*Prescriber characteristics*: *gender*, *age*, board certification, *hospital affiliation*, per cent of working hours spent in a hospital setting, clinical research experience (total number of clinical studies, *number of clinical studies conducted for the sponsoring company, number of clinical studies conducted in the therapeutic class of the new drug*, number of clinical studies with the compound), relative grant amount received by investigator, *pre-launch prescribing volume, pre-launch prescribing volume in the therapeutic class of the new drug, pre-launch prescribing of the sponsoring company’s products as a percentage of total prescribing (company loyalty)*, market share of the sponsoring company 3 and 6 months post launch as percentage of total prescribing volume
				*Drug/market characteristics*: therapeutic novelty, number of drugs tested for the same indication, participation of a contract research organisation, *market share 1 year post launch*
Glass and Rosenthal 2004 [28]	3646 doctors, US	32 new drugs	binomial logistic regression	*Prescriber characteristics*: gender, *age*, *board certification*, *speciality*, *clinical investigation experience*, *hospital affiliation*, *pre-launch prescribing volume*, *pre- launch prescribing volume in the same therapeutic class as the new drug*, *pre-launch company loyalty*
				*Drug characteristics*: therapeutic novelty, *marketing budget of the pharmaceutical company assigned for the new drug*
Glass and Rosenthal 2005 [29]	2287 clinical trial investigators, US	38 new drugs	ordinary least squares (OLS) and binomial logistic regression	*Prescriber characteristics*: gender, *age*, board certification, *speciality*, *hospital affiliation*, *percentage of working hours spent in a hospital setting*, clinical research experience (*total number of clinical trials*, total number of clinical studies conducted, *total number of clinical studies conducted for the sponsoring company*, *total number of clinical studies conducted in the therapeutic class of the new drug*, total number of clinical studies conducted with the compound), total pre-launch prescribing volume, *pre-launch prescribing volume in the therapeutic class of the new drug*, *pre-launch prescribing volume of sponsoring company’s products*, *pre-launch prescribing volume of sponsoring company’s products as a percentage of total prescribing (company loyalty)*
				*Drug characteristics*: *market share of the new drug 12 months post launch*, *marketing budget of the pharmaceutical company assigned for the new drug*
Glass and Dalton 2006 [30]	484 phase IV clinical trial investigators, US	new drugs for the outpatient treatment of 8 disorders or diseases	binomial logistic regression	*Prescriber characteristics*: gender, *age*, board certification, speciality, practice type (office, hospital, academic medical centre, other), per cent of working hours spent in a hospital setting, clinical research experience (number of clinical trials conducted, number of clinical studies conducted for the sponsoring company, number of clinical studies conducted in the same therapeutic class as the new drug, number of clinical studies conducted with the compound), relative grant amount received, *pre-study prescribing volume*, *pre-study prescribing volume in the same therapeutic class as the new drug*, *pre-study prescribing volume of the new drug*, pre-study prescribing volume of sponsoring company’s products, *pre-study prescribing volume of sponsoring company’s products as a percentage of total prescribing (company loyalty)*
				*Drug characteristics*: market share of the new drug in its therapeutic class 12 months post launch, *revenue of the pharmaceutical company*
Greving et al. 2006 [11]	70 GPs, 9470 hypertensive patients, the Netherlands	1 new drug (angiotensin II receptor blocker (ARB))	multilevel logistic regressions	*Prescriber characteristics*: *use of commercial information*, use of medical journals, CME, use of other professional information, *use of a prescribing decision support system*, *personal involvement in PTAMs*
				*Practice characteristics*: type (solo or group/partnership), *location (urban or rural)*
				*Patient characteristics*: *age*, gender, number and type of comorbidities, *referrals to internist/cardiologist*
				*Drug characteristics*: perceived benefits (survey data)
JP Griffin and TD Griffin 1993 [31]	10 developed countries	drugs introduced in the last 5 years	descriptive statistics	*Prescriber characteristics*: *nationality*
Groves et al. 2010 [6]	925 doctors and all their prescriptions, Canada	4 new drugs (COX-2 inhibitors)	correlation analysis with t-tests	*Prescriber characteristics*: *gender*, *age*, birthplace, *speciality*, training location (domestic or overseas qualification), *professional age*, *prescribing volume of four related drugs*
				*Practice characteristics*: *location (urban or rural)*
Helin-Salmivaara et al. 2005 [32]	2558 doctors, 507262 prescriptions from the same therapeutic class, Finland	2 new drugs (celecoxib and rofecoxib)	general linear mixed model	*Prescriber characteristics: gender*, professional age, *speciality*
				*Patient characteristics*: *age*, gender
Huskamp et al. 2013 [33]	30369 doctors, US	9 new drugs (second-generation antipsychotics)	Cox’s proportional hazard model	*Prescriber characteristics*: *gender*, *age*, *speciality*, hospital affiliation*, *prescribing volume in the same therapeutic class as the new drug*, *training location (domestic or overseas qualification, top- or non-top-25 medical school)*
				*Practice characteristics*: *type (solo or group/partnership)*
				*Drug characteristics*: *degree of innovation (original formulation or reformulation)*
Inman and Pearce 1993 [34]	3346 GPs, England	27 new drugs	descriptive statistics	*Prescriber characteristics*: *gender*, *number of treatments*, *professional age*, *training location (domestic or overseas qualification)*
Iyengar et al. 2011 [12]	185 doctors, US	1 new drug, third entry in the category (for treatment of viral infections)	discrete-time hazard model	*Prescriber characteristics*: speciality (GP or SP), number of patients, referral to other doctors, *prescribing volume in the same therapeutic class as the new drug*, self-reported leadership*, *detailing*, *indegree centrality (referral, discussion, and total network)*, outdegree centrality (referral, discussion, and total network), peer influence through adoption and usage*
				*Practice characteristics*: type (solo or group/partnership), type of hospital (university/teaching), location (city dummies)
Kozyrskyj et al. 2007 [35]	12 million patients and 2000 doctors, Canada	4 new drugs, from 4 therapeutic classes	polytomous logistic regression	*Prescriber characteristics*: age, gender, speciality*, professional age, *training location*, hospital affiliation*
				*Practice characteristics*: type (solo or group/partnership)
				*Patient characteristics*: age, gender, neighbourhood income quintile*, prescription reimbursement status, presence of chronic conditions
Lin et al. 2011 [36]	155 SPs (psychiatry) affiliated with 12 healthcare centres, Taiwan	1 new drug (antidepressant, in the selective norepinephrine reuptake inhibitor (SNRI) family)	Cox’s proportional hazard model	*Prescriber characteristics*: age, gender, *speciality (in depressive disorder)*, hospital experience (number of current workplaces), outpatient services (number of visits), *peers’ adoption ratio*, *opinion leaders’ adoption ratio,* (dis)similarities with adopting peers’ *age*, gender, and *tenure*, *SNRI proportion (past experience and preference for SNRI)*
				*Practice characteristics*: size (number of SPs), *ownership (public or private)*, *location*
Liu et al. 2011 [37]	41488 patients, 4429681 prescriptions, Taiwan	7 new drugs (oral hypo-glycemic agents, for treatment of diabetes)	logit model	*Practice characteristics*: *market share (number of outpatient visits)*, ownership* (public or private not-for-profit or private for-profit), *accreditation level (clinic, academic medical centre, metropolitan hospital, or local community hospital)*, location
				*Patient characteristics*: age, gender, *disease severity*, *number of prescriptions per visit*
				*Drug characteristics*: *prescribing volume in the same therapeutic class as the new drug*
Liu and Gupta 2012 [13]	2129 doctors, US	1 new drug (for treatment of a chronic condition)	discrete-time hazard model	*Prescriber characteristics*: *speciality*, *prescribing volume in the same therapeutic class as the new drug*, *number of detailing visits*, *number of medical meetings and events*, *number of patient requests made to doctors*, *number of peers in geographic proximity who had already adopted the new drug*
				*Drug characteristics*: *journal advertising*, *linear time trend*
				*Patient characteristics*: median age of people in the community, *average household income*, *health insurance index*, *percentage of white population*
Manchanda et al. 2008 [38]	466 doctors, Manhattan (New York City), US	1 new drug (for treatment of a chronic condition)	discrete-time hazard model	*Prescriber characteristics: contemporaneous effect of detailing*, *accumulated stock of detailing*, *accumulated stock of sampling*, *contagion measure (number of adopting doctors in geographic proximity)*
				*Drug characteristics*: aggregate marketing expenditure (direct-to-consumer advertising (DTCA)), *time since launch*
Mark et al. 2002 [14]	187 doctors, 752 patients, prescriptions from medical records, US	4 new drugs (antipsychotics—(clozaril, risperidone, olanzapine, and quetiapine)	bivariate and multivariate probit regression analysis	*Prescriber characteristics*: age, gender, board certification*, number of patients*, number of contacts with pharmaceutical representatives*, attendance on professional meetings*, preference for atypical initial treatments, preference for atypical treatments for nine different conditions*, percentage of patients influenced by individual medication costs
				*Practice characteristics*: location
				*Patient characteristics*: *age*, *gender*, *race/ethnicity*, education, marital status, insurance status, *first onset of the disorder*, diagnosis*, *symptoms*, hospitalisation in the past 12 months*
Mizik and Jacobson 2004 [39]	74075 doctors, US	1 new drug, within a well-established therapeutic area, and 2 older drugs	dynamic fixed effects distributed lag regression	*Prescriber characteristics*: speciality*, *detailing volume*, *sampling volume*, prescribing volume in the same therapeutic class as the new drug (competitor prescribing)
Ohlsson et al. 2009 [5]	73547 doctors, 32011 patients, Sweden	1 new drug (rosuvastatin, for treatment of high blood cholesterol)	generalised estimation equations and alternating logistic regression	*Practice characteristics*: *ownership (public or private)*, *proximity to SPs*, location (urban or rural), size (prescribing volume)
				*Patient characteristics*: *age*, *gender*, *income*, *marital status*, birthplace, length of residence in Sweden
Steffensen et al. 1999 [40]	319 GPs, 193876 prescriptions, Denmark	5 generically new compounds	multiple logistic regression	*Prescriber characteristics*: *gender*, age
				*Practice characteristics*: *type (solo or group/partnership)*, *size (number of patients)*, number of consultations per patient, number of telephone consultations per patient, number of home visits per patient, *number of procedures performed per patient*, number of laboratory tests performed per patient
Ruof et al. 2002 [41]	72 GPs, 28 SPs (neurology), Germany	1 new drug class (for treatment of Alzheimer’s disease)	Sperman’s rank correlation coefficient	*Prescriber characteristics*: *speciality (GP or neurologist)*
				*Drug characteristics (perceived)*: safety, efficacy, life quality improvement, nursing home admission delayed, budgetary impact
Tamblyn et al. 2003 [1]	1661 doctors, 669867 elderly patients, Canada	20 new drugs, from 6 therapeutic classes	multivariate logistic and conditional Poisson regressions	*Prescriber characteristics*: *gender*, *speciality*, professional age*, *training location*
				*Practice characteristics*: *location (urban or rural)*, referral rate to SPs who prescribe drugs in the same therapeutic class as the new drug*, *size*
				*Drug characteristics*: detailing (minutes), advertising (pages)
				*Patient characteristics*: *proportion of elderly patients in the practice population*
Van den Bulte and Lilien 2001 [15]	121 GPs, four small cities in Illinois, US	1 new drug (tetracycline, a broad-spectrum antibiotic)	discrete-time hazard model	*Prescriber characteristics: professional age*, *number of journals read, position*, *scientific orientation*, status (number of nominations received as advisor or discussant—2 contagion variables to capture word of mouth operating over direct ties and 2 contagion variables to capture competition for status between structurally equivalent doctors
				*Drug characteristics*: seasonal effect, *depreciation-adjusted stock of marketing effort by the first entrant*, depreciation-adjusted stock of marketing effort by the two subsequent entrants

In alphabetical order, by first author. Variables in italics without asterisk: significant impact on new drug uptake in all specifications of the study. Variables in italics with asterisk: significant impact on new drug uptake in some specifications of the study.

### Study characteristics

#### Sample populations

The 35 eligible studies were all conducted in developed countries—mostly in North America (17 studies) [1, 6, 7, 12–15, 19, 26–30, 33, 35, 38, 39] and North-Western Europe (11 studies) [5, 11, 18, 20–23, 32, 34, 40, 41]—14 studies were conducted in the US [7, 12–15, 19, 26–30, 33, 38, 39], four in Denmark [20–22, 40], three each in Canada [1, 6, 35] and Spain [16, 24, 25], two each in the Netherlands [11, 23] and Taiwan [36, 37], and one each in Australia [17], Finland [32], Germany [41], Ireland [18], Sweden [5], and the UK [34], while one study [31] covered ten developed countries—Belgium, Canada, France, Germany, Italy, Japan, the Netherlands, Spain, the UK, and the US.

The sample populations varied greatly, in both unit of observation and size. In one study [31], the unit of observation was the country—in five others [5, 16, 20, 21, 37], the healthcare provider, ranging from 32 healthcare centres [16] to 191 practices [20, 21], with a mean of 146 and one study not disclosing the number [37]. However, doctors were the unit of observation for 29 [1, 6, 7, 11–15, 17–19, 22–30, 32–36, 38–41] of the 35 eligible studies—nine studies observed general practitioners (GPs) [7, 11, 15, 17, 18, 22, 23, 34, 40], three specialists (SPs) [14, 25, 36], and 17 both [1, 6, 12, 13, 19, 24, 26–30, 32, 33, 35, 38, 39, 41]. The sample populations ranged from 68 [22] to 74,075 doctors [39], with a median of 770.

#### Study drugs

The study drugs covered a wide range—antibiotics, cardiovascular drugs, coxibs, antihypertensives, and antidepressants, for example. Ten studies used the prescription data for one drug/therapeutic class [5, 7, 11–13, 15, 36, 38, 39, 41], eight studies used the prescription data for two to five drugs [6, 14, 17, 22, 23, 32, 35, 40], six for six to ten drugs [18, 25, 26, 30, 33, 37], and 11 for more than ten drugs [1, 16, 19–21, 24, 27–29, 31, 34]—50 new drugs [16] and 72 new compounds of varying therapeutic novelty [27] were the maxima. Twenty two studies assessed highly innovative drugs [5, 6, 11, 13, 14, 17, 20–22, 26, 27, 29–33, 35–38, 40, 41], three me-too drugs [7, 12, 15] and ten both [1, 16, 18, 19, 23–25, 28, 34, 39].

#### Methods

Eight studies used simple statistical methods [6, 7, 17, 20, 26, 31, 34, 41]—comparison of means or association or correlation analyses, for example. Other eight studies—published mostly in the 2010s—used either discrete-time hazard models or very similar survival analyses [12, 13, 15, 18, 25, 33, 36, 38]. However, the most popular methods of assessing the importance of particular variables were logistic and linear regressions, used in 11 [1, 5, 11, 14, 23, 28–30, 35, 37, 40] and respectively eight studies [16, 19, 21, 22, 24, 27, 32, 39].

#### Dependent variables

The dependent variables under consideration varied greatly, with some studies focusing on either prescriber [17, 22, 23, 26, 31, 34, 39] or practice [16, 21] characteristics, whilst others assessing two or more categories of variables [1, 5–7, 11–15, 18–20, 24, 25, 27–30, 32, 33, 35–38, 40, 41]—prescriber and practice characteristics were the most common such combination. On average, the studies included nine dependent variables. Table 3 summarises the dependent variables found significant in at least one of the 35 eligible studies, whereas the last column of Table 2 details all the inputted variables—their number highlights the complexity of new drug diffusion.

**Table 3:** **Summary of characteristics influencing new drug diffusion**

Prescriber characteristics (micro- and meso-level)	Practice characteristic (meso-level)	Drug characteristics (meso-level)	Patient characteristics (meso-level)
**Socio-demographic characteristics (micro-level)**	Location (urban or rural) (3/7)	Marketing budget of the pharmaceutical company assigned for the new drug (5/7)	Age (6/9)
Gender (7/15)	Type (solo or group/partnership) (4/7)	Overall acceptance (5/6)	Gender (1/6)
Age ( 9/14)	Size (2/6)	Therapeutic novelty (2/3)	Health (3/4)
Professional age (4/5)	Ownership (private or public), management (reformed or non-reformed), and orientation (for profit or not for profit) (3/4)	Competition (1/1)	Socioeconomic characteristics (income, education, and health insurance) (3/4)
Training location (4/5)	Region (1/4)		Marital status (1/2)
Number of current workplaces (1/2)	Accreditation level (1/2)		
Nationality (1/1)	Diagnostic and therapeutic activities (2/2)		Race/ethnicity (2/2)
**Scientific orientation (micro-level)**	Employee composition (1/2)		
Speciality (10/16)	Other (2/2)		
Hospital affiliation (4/8)			
Board certification (2/6)			
Clinical trial participation (3/3)			
CME [continuing medical education] and pharmacotherapy audit meetings (PTAMs) (2/3)			
Number of professional journals read (2/3)			
Perceived scientific orientation (2/3)			
Specialist meetings and events (2/3)			
Position (1/1)			
**Prescribing characteristics (meso-level)**			
Prescribing volume in the therapeutic class of the new drug (10/11)			
Total number of patients/prescriptions (6/9)			
Prescribing volume of drugs by the same pharmaceutical company (4/4)			
Portfolio width (1/1)			
**Marketing efforts targeted at doctors (meso-level)**			
Detailing (4/6)			
Sampling (2/2)			
Direct-to-consumer advertising (DTCA) (1/1)			
**Contagion through social networks (meso-level) (5/6)**			

In brackets, the number of studies where the variable was found significant in the adoption process over the number of studies assessing the impact of the variable.

### Factors affecting new drug uptake

In both primary and secondary care, new drug diffusion is subject to interacting influences. The idea that early prescribers do not generally exist [20, 35, 40] does not necessarily mean that adoption of new drugs is random. Rather, adoption varies across prescribers, with the prescriber, patient, practice, and drug characteristics. The 35 eligible studies [1, 5–7, 11–41] identified several—partly overlapping—characteristics that proved crucial in the adoption process, and that predicted—seemingly consistently—new drug uptake. This review will clearly indicate the characteristics constant across drug types. However, in a number of cases, there is contradiction within the literature. Whilst some studies found one particular variable significant, others found no evidence for the predictive power of that variable. Also, reported coefficients between one particular variable and new drug uptake were not always consistent in terms of sign. These anomalies will also be clearly indicated in this review.

#### Prescriber characteristics

Twenty nine of the 35 eligible studies investigate at least one prescriber characteristics [1, 6, 7, 11–15, 18, 19, 22–36, 38–41].

### Socio-demographic characteristics

Nineteen of the 35 eligible studies investigate at least one socio-demographic characteristics of prescribers [1, 6, 7, 14, 15, 18, 19, 24, 27–36, 40].

*Gender* (n = 15) [1, 6, 14, 19, 24, 27–30, 32–36, 40]. Gender played an influential role in the early adoption of new drugs in seven [1, 6, 27, 32–34, 40] of the 15 studies—in the other eight [14, 19, 24, 28–30, 35, 36], the variable was not significant. Where gender was found influential, male prescribers were much more likely to adopt new drugs than female prescribers, a finding consistent across drug types. In a large-scale quantitative study of British doctors, the first of its kind, Inman and Pearce [34] observed that male doctors had much higher rates of new drug utilisation than female doctors—in the group that prescribed new drugs most heavily, women accounted for only 9 per cent. Later studies came to similar conclusions [1, 6, 27, 32, 33, 40]. In contrast, Glass [27] found that women were somewhat more likely than men to embrace new drugs six months after launch, but not after just three.

*Age* (n = 14) [6, 7, 14, 18, 19, 24, 27–30, 33, 35, 36, 40]. Age was associated with new drug uptake in nine studies [6, 7, 18, 27–30, 33, 35]. Seven of these found that early prescribers were younger than the majority [7, 18, 27–30, 33], with younger doctors’ higher propensity for innovation and older doctors’ more established prescribing practices as the most likely explanations, whereas the other two found that early prescribers were likely to be older [6, 35]. Five other studies found no correlation at all [14, 19, 24, 36, 40].

*Professional age* (n = 5) [1, 6, 15, 34, 35]. Defined as the number of years since graduation from medical school [15, 34], professional age can be proxied by the number of years spent in practice [6, 35]. Although professional age evidently correlates highly with age^a^, its impact on new drug uptake is less obvious—one study found no association [35], two studies found significant positive associations [6, 34], whereas two others found significant negative associations [1, 15]. However, positive associations were found in less powerful, bivariate analyses [6, 34]. In more powerful, multivariate analyses, similar to studies assessing the impact of age on new drug uptake, younger professional age was found favourable to early adoption [1, 15].

*Training location* (n = 5) [1, 6, 33–35]. So far, due to data constraints, only five studies assessed the role of training location in new drug uptake [1, 6, 33–35], and—with one exception [6]—found it influential. From British [34] and Northern American [35] perspectives, more new drugs were prescribed by doctors with overseas qualifications than by doctors with domestic qualifications. At the same time, Huskamp et al. [33] reported that doctors who had graduated from a top-25 medical school were slower to adopt than others, with high-ranking medical schools possibly more conservative than others in adopting new drugs and granting exposure to pharmaceutical marketing. Also, Tamblyn et al. [1] found that doctors who had graduated from the youngest medical schools had higher relative rates of new drug use than others.

*Number of current workplaces* (n = 2) [24, 36]. The two studies led to ambiguous conclusions—García et al. [24] found that doctors with more than one workplace adopted new drugs earlier than others, whereas Lin et al. [36] found the number of current workplaces irrelevant.

*Nationality* (n = 1) [31]. The study where the speed of adoption was measured across nationalities found that doctors in Italy, Germany, Spain, and the US were more likely to prescribe products with new active chemical entries than doctors in the other six countries under review, Belgium, Canada, France, Japan, the Netherlands, and the UK [31]. British doctors were found least likely to prescribe new drugs—they relied on progressively fewer active substances for a greater proportion of their prescriptions.

### Scientific orientation

Twenty four of the 35 eligible studies access some aspects of prescribers’ scientific orientation [1, 6, 7, 11–15, 19, 22–30, 32, 33, 35, 36, 39, 41].

*Speciality* (n = 16) [1, 6, 7, 12, 13, 24, 25, 28–30, 32, 33, 35, 36, 39, 41]. Intuitively, doctors are likely to prescribe new drugs in clinical and therapeutic areas where they feel familiar or have special interests. Also intuitively, since a significant amount of general practice prescribing is hospital-initiated or hospital-led [42, 43], SPs are likely to influence new drug uptake among GPs, through advice or example. There is a broad consensus in the literature that new drugs diffuse into general practice in two stages, with SPs as innovators and GPs as followers [44]. In line with this model, ten of the 16 eligible studies reported faster adoption among SPs in secondary care than among GPs in primary care, and found that specialisation in the therapeutic areas of newly introduced drugs associated with adoption rate significantly and positively [1, 7, 13, 25, 28, 29, 32, 33, 36, 41]. For example, Garjón et al. [25] analysed the diffusion of eight new drugs suitable for common disorders in both primary and secondary care concluded that SPs adopted new drugs earlier than GPs^b^. There is also a broad consensus in the literature that SPs adopt new drugs in their speciality earlier than GPs and other SPs, both of whom tend to rely on norms—low prescribing proportions do not justify high cognition time costs [33].

However, six of the 16 eligible studies came to different conclusions [6, 12, 24, 30, 35, 39]. Groves et al. [6] found that GPs were more likely early prescribers than SPs. Kozyrskyj et al. [35] found mixed evidence—in some cases, GPs adopted new drugs earlier than SPs, whereas in others no significant associations were observed. Four other studies found no or weak associations between speciality and new drug uptake [12, 24, 30, 39].

*Hospital affiliation* (n = 8) [19, 22, 27–30, 33, 35]. Association with an academic centre such as a university or a research institute—through teaching, publishing, or holding an academic appointment, for example—suggests a professional orientation that may result in early adoption of new drugs. However, hospital-affiliated doctors face formulary restrictions.

In line with the heightened professional orientation argument, two of the eight eligible studies found that doctors in hospital settings adopted some new drugs earlier than doctors with no hospital affiliation—two out of four new drugs, according to Kozyrskyj et al. [35], and two out of nine, according to Huskamp et al. [33]. In contrast, four studies reported that office-based clinical trial investigators were more likely to prescribe newly marketed drugs than other doctors [19, 27–29]. Moreover, the longer doctors worked in hospitals, the less likely they were to prescribe new drugs [19, 29], not least due to hospital formularies [28, 29]. Two studies observed no difference between hospital-affiliated and office-based doctors [22, 30].

*Board certification* (n = 6) [14, 19, 27–30]. In the US, board certification quantifies on a voluntary basis doctors’ mastery of the core body of knowledge and skills in their chosen specialities at a specific time [45]. Board certification was found consistently associated with adoption in two [19, 28] of the six studies, but not in the others [14, 27, 29, 30].

*Clinical trial participation* (n = 3) [19, 26, 28]. Due to proximity to research and understanding of the evidence base, clinical trial participation increased early adoption of new drugs and had a significant and positive impact on subsequent prescribing in all eligible studies [19, 26, 28]. Other studies published within the same research framework by Glass and colleagues provided further insight into the new drug uptake of clinical trial investigators [27, 29, 30]. As expected, participation in drug class clinical trials was also associated with increased prescribing of the study drug [27, 29]. Against intuition, the higher the number of clinical studies conducted in total [27], or for the sponsor [27, 29], the lower doctors’ sense of identification with a new drug and the lower the likelihood of prescribing it.

*CME and pharmacotherapy audit meetings (PTAMs)* (n = 3) [11, 22, 23]. Dybdahl et al. [22] found that CME was significantly and positively associated with new drug uptake in some clinical areas—however, the association was weak and inconsistent across drug classes. Greving et al. [11] found no relationship between CME and new drug uptake and reported negative relationships between doctors involved in PTAMs and new drug uptake. Florentinus et al. [23] did not analyse the differences in new drug uptake between GPs who attended PTAMs voluntarily^c^ and those who opted out, but they did analyse the effects of PTAMs on early new drug prescribing by GPs. GPs who attended low-quality PTAMs—without structure or concrete outcomes—were more than twice as likely to prescribe new drugs early post-marketing than those who attended high-quality PTAMs—with frequent concrete outcomes and outcome evaluations. The authors argued that high-quality PTAMs may restrict the number of new drugs GPs can prescribe, or may foster a rather negative attitude towards prescribing new drugs, but found no empirical evidence. Moreover, the authors found that increases in the number of GPs—and pharmacists—attending PTAMs led to increases in new drug prescribing by GPs. These findings underscore the social network aspect of such forums, which facilitate interactions among healthcare professionals. Doctors who attend more or larger forums interact and influence one another’s prescribing more than they would otherwise. The section ‘Contagion through social networks’ discusses the role of social networks in new drug uptake in further detail.

*Number of professional journals read* (n = 3) [7, 11, 15]. A possible measure of scientific orientation, the number of professional journals doctors read is a variable quantifiable only through survey. Peer-reviewed journals are important sources of information on new drugs [8, 43, 46]. Sound research evidence is considered very influential in reaching prescribing decisions [42, 43].

Three studies assessed the impact of professional journals on new drug uptake [7, 11, 15]. Two of these used the same dataset and reported that the higher the number of journals read, pharmaceutical company newsletters and scientific and professional publications included, the earlier doctors adopted new drugs [7, 15]. However, Greving et al. [11] concluded that new drug adoption among GPs was still determined more by promotional than professional information—no significant relation was observed between the use of scientific medical journals and new drug uptake^d^.

*Perceived scientific orientation* (n = 3) [7, 15, 22]. Coleman et al. [7] measured doctors’ perceived scientific orientation—through (dis)agreement with the statement ‘it is more important for a physician to keep himself informed of new scientific developments than to devote more time to his patients’—and found that profession-oriented doctors used new drugs earlier than patient-oriented doctors, a conclusion confirmed by Van den Bulte and Lilien’s [15] reanalysis. In contrast, Dybdahl et al. [22] found no clear association between self-rated GP clinical interest and new drug uptake.

*Specialist meetings and events* (n = 3) [7, 13, 14]. Specialist meetings, congresses, conferences, and symposia contribute to professional development—by providing a highly valued source of information and acting as catalyst for early awareness and positive evaluation, such forums may accelerate the early adoption of new drugs. In their classic study, Coleman et al. [7] found that doctors who attended many out-of-town speciality meetings—or who conscientiously attended conferences in their own hospitals—were more likely to innovate than others. Non-speciality meetings and county medical society meetings had no influence over new drug uptake. In a recent, carefully designed study, Liu and Gupta [13] also reported that attending professional events had a positive impact on new drug uptake. Mark et al.’s [14] bivariate analysis found that doctors who had attended a professional meeting within the past five years were more likely to innovate. Their multivariate analysis, however, found that attending professional events did not influence new drug prescription significantly.

*Position* (n = 1) [15]. Van den Bulte and Lilien [15] found that hospital doctors in managerial or honorary positions adopted new drugs later than others, due to limited involvement in actual medical practice.

### Prescribing characteristics

Fifteen of the 35 eligible studies investigate at least one prescribing characteristics [6, 7, 12–14, 18, 19, 24, 27–30, 33, 34, 36].

*Prescribing volume in the therapeutic class of the new drug* (n = 11) [6, 7, 12–14, 27–30, 33, 36]. Ten of the 11 studies found that the higher the prescribing volume in the therapeutic class of a new drug, the higher the likelihood of early adoption of that new drug [6, 7, 12–14, 28–30, 33, 36]. Although Iyengar et al. [12] found supporting empirical evidence for only one of two drugs, the variable is likely to remain significant when summing up the prescribing volumes of the two drugs. Mark et al. [14] used stated preference for new medication within the drug class as variable and confirmed the relationship in the bivariate model, but not in the multivariate model—an insufficiently powerful result. Against mainstream research, Glass [27] reported that doctors with higher prescribing volumes in the therapeutic class of the study drug were more reluctant to prescribe the study drug, once on the market, possibly due to strong preferences for other drugs.

*Total number of patients/prescriptions* (n = 9) [12, 14, 19, 24, 27–30, 34]. To address the unfulfilled medical needs of some of their patients, doctors with high patient flows seem particularly alert to new drugs. Six of the nine studies reported that the higher the total prescribing volume, the higher the likelihood of early adoption of new drugs [19, 24, 27, 28, 30, 34] ^e^. In contrast, the other three studies showed no relationship between the number of patients/prescriptions and new drug uptake [12, 14, 29]. However, Glass and Rosenthal [29] found that once doctors had written at least one prescription for the study drug, the total number of pre-study prescriptions became a significant variable. Also, Mark et al.’s [14] bivariate model found that the more schizophrenia patients doctors were treating, the earlier they adopted atypical medication—the variable became an insignificant determinant of new drug uptake only in the multivariate analysis.

*Prescribing volume of drugs by the same pharmaceutical company* (n = 4) [27–30]. Unambiguously, all four studies found that the higher the prescribing volume of drugs by the same pharmaceutical company, the higher doctors’ likelihood of early adoption of other drugs by that pharmaceutical company [27–30]. The authors argued that doctors are likely to prescribe new drugs if already writing high numbers or large percentages of prescriptions from the sponsoring company—either due to increased detailing by that pharmaceutical company or to doctors’ confidence and trust in that company or in the company’s sales representatives [27–30]. The high explanatory power of this variable is evidently related to the marketing efforts of pharmaceutical companies discussed in the section ‘Marketing efforts targeted at doctors’.

*Portfolio width* (n = 1) [18]. Bourke and Roper [18] defined portfolio width as the percentage of drugs prescribed by individual GPs out of the portfolio of drugs prescribed by all the GPs in that practice. Most probable, this variable correlates highly with practice size—the more patients doctors treat, the more likely they are to require a larger portfolio of drugs from which to prescribe. The authors found that the time to adoption was decreasing substantially with increasing portfolio widths, for all six study drugs. A 1 per cent increase in a GP’s portfolio width decreased time to adoption between three and 10.5 months, the variable having very high explanatory power.

### Marketing efforts targeted at doctors

Pharmaceutical companies typically direct their marketing efforts at doctors and—of late—patients. Several studies reported that doctors had negative to neutral attitudes towards promotional efforts targeted at them and tried to minimise the importance of sales representatives, to avoid distorted, selective, and overly positive information [47–49]. Nevertheless, detailing (personal selling through sales representatives) is an inexpensive and convenient source of information. Generally, GPs indicated greater preference for commercial information than SPs—time constraints and the broader range of conditions they treated did not allow them to review satisfactorily all relevant professional information [8, 49, 50].

The marketing efforts directed at doctors comprised detailing, sampling (provision of drugs free of charge), doctor meetings and events, and advertisements in medical journals [48, 51]. They aimed to boost profits by incorporating new drugs early in their lifecycles, by raising awareness among top professionals, and by maintaining the new drugs’ first-choice statuses within their respective therapeutic groups [52]. Pharmaceutical marketing not only raises awareness—it evidently influences decision making too. Six of the 35 eligible studies investigate some aspects of the marketing efforts targeted at doctors [7, 12–14, 38, 39]. The empirical evidence presented below shows that pharmaceutical promotion affected prescription behaviour positively, albeit to various degrees.

*Detailing* (n = 6) [7, 12–14, 38, 39]. Historically, detailing has been the primary promotional instrument of the pharmaceutical industry—one third of total marketing expenditure has been directed at detailing [51]. Four of the six studies reported that doctor-targeted detailing affected early adoption of new drugs significantly and positively—doctors tended to respond to both current and past detailing positively [12, 13, 38, 39]. In contrast, the other two studies found no empirical evidence for the positive impact of detailing [7, 14]. However, Burt [53] used the same dataset as Coleman et al. [7] and found a significant association, possibly due to methodological differences. Studies reporting positive influences used sales call data from pharmaceutical company records, whereas studies reporting no empirical evidence used survey data—norm-conforming doctors with negative attitudes towards sales representatives may have reported less contact than they actually had.

The marketing literature has documented well the impact of pharmaceutical promotions on doctors’ choices, including through extensive reviews [48, 54]. Early studies used aggregate data [55–58], and almost all studies found a strong positive effect of detailing on sales [48, 54]. Recent research used behavioural data [59–61] and came to the broad consensus that detailing affects the number of prescriptions for new drugs positively and significantly. This effect was robust to a wide variety of model specifications and datasets [48, 54]. To provide a more complete picture of sales and prescribing behaviours, several recent models added numerous other marketing variables and found that detailing had a positive and significant effect on sales, even after controlling for other marketing mix instruments [48, 54]. Most studies also found that—relative to other marketing instruments—detailing had the largest effect^f^.

*Sampling* (n = 2) [38, 39]. Both studies found that the volume of samples that had been left with doctors in the past had a significant and positive impact on adoption behaviour [38, 39], even if the impact was very small [39]. These findings were in line with the overarching conclusion of the drug marketing literature [59, 60].

*Direct-to-consumer advertising (DTCA)* (n = 1) [13]. If allowed, DTCA in the mass media may influence early adoption of new drugs through patient requests targeted at doctors. In general, due to time constraints and the desire to avoid conflict and increase patient role in decision making, doctors honour patient requests [49]. The eligible study found that the volume of patient requests for a new drug had a positive and significant impact on doctors’ adoption decisions [13], and the extant marketing literature contributed to a more complete understanding of how DTCA impacted the demand for new drugs. Two rigorous literature reviews concluded that DTCA was associated with increased prescribing of advertised products, due to substantial impact on patients’ requests for specific drugs and doctors’ prescribing confidence [54, 62]. When detailing was controlled for, promotions aimed directly at doctors turned out to affect prescription choice much more than promotions aimed at consumers [54, 63–66]. DTCA had only a short-run effect on choice, whilst the effect of detailing wore out slowly, over the course of the year. There is a general consensus in the literature that DTCA is effective in generating foot traffic into doctors’ offices and increasing the aggregate drug demand per therapeutic class, without affecting doctors’ prescribing choices within the therapeutic class [64, 66, 67]. Once patients get to see their doctors, detailing and sampling have the most significant effect on what drugs doctors choose [64–66] ^g^.

Pharmaceutical companies may facilitate new drug awareness in many other ways, all with a substantial promotional component—for example, funding of clinical trials, sponsoring of conferences and CME, and journal advertisements. Kremer et al. [54] reported that the elasticities of these other direct-to-doctor instruments are significant, but modest in size. This review discusses the impact of these other—partly scientific, partly promotional—efforts in relation with variables measuring doctors’ scientific orientation (see the section ‘Scientific orientation’).

### Contagion through social networks

When new drugs are launched, doctors’ behaviours are affected by other doctors’ knowledge, attitudes, and behaviours, thus reducing safety and efficacy uncertainties. Interpersonal communication between opinion-leading doctors and peers seems critical to fast—and wide—acceptance of innovative drugs [8, 50, 68]. Personal contacts may provide real stimuli, since key opinion leaders present reliable, easy-to-digest assessments of new drugs. While other sources of information provide the nurturing groundwork of necessary knowledge, behavioural change requires the legitimising power of personal advice from informed and respected colleagues [69].

The main findings of the six eligible studies [7, 12, 13, 15, 36, 38] are summarised below, in chronological order—five of them found empirical evidence for contagion, even after controlling for a wide variety of factors.

In their path-breaking and highly cited *Medical Innovation: A Diffusion Study*, *Coleman et al.* [7] argued that the network of informal relations among doctors was highly effective in transferring information and influencing the diffusion of pharmaceutical innovations^h^. Doctors’ adoption decisions were affected by interactions with other doctors—socially integrated doctors introduced new drugs quicker than their more isolated colleagues. The finding was found valid for all three social structures of the medical community studied—advisor, discussion, and friendship networks. However, doctors’ professional interactions had a larger effect on adoption time than social interactions. Moreover, the channels of influence among doctors operated most powerfully during the first few months after the release of a new drug.

*Van den Bulte and Lilien* [15] found that contagion effects disappeared when journal advertising was controlled for—first entrants’ marketing efforts, not social contagion, were the dominant driver in increasing doctors’ adoption hazard with time. The authors explicitly underscored the importance of controlling for potential confounds, such as marketing efforts, when studying the role of social contagion in new drug diffusion. In a subsequent working paper, deemed ineligible because it did not meet the first eligibility criteria, they used new event history models for a two-stage adoption process—awareness followed by evaluation and adoption [70]. When marketing efforts were allowed to affect awareness and social contagion was allowed to affect evaluation, both effects became statistically significant. In the presence of strong mass media effects, the patterns of social contagion were weak—contagion had a significant effect in the second stage.

In their carefully designed study, *Manchanda et al.* [38] found that doctors’ decisions to adopt were influenced positively by contagion—that is, doctors’ probabilities to adopt increased significantly with increases in fellow doctor adoption. The authors determined the social networks of doctors on basis of geographic proximity, defined as the area within a 20-mile radius around a doctor’s office. The observed positive and significant contagion effect persisted even after controlling for time trends and marketing efforts. The authors ruled out many alternative explanations, including uniqueness of the social networks under analysis or unobserved common variables that affect all the network members similarly—for example, sales representatives or institutional factors such as working for the same hospital, using the same formularies, and attending the same meetings and events sponsored by pharmaceutical companies.

Interestingly, Manchanda et al. [38] had also calculated the proportion of adoption probability arising from marketing activity relative to that arising from contagion and had found that marketing played a larger role in adoption earlier on. However, consistent with Iyengar et al. [12] and Narayanan et al. [65], contagion dominated from month 4 onwards, asymptoting to around 90 per cent of the effect by month 17.

In a partly similar setting, *Liu and Gupta* [13] also found that the estimated effect of contagion among doctors in geographic proximity was positive and significant.

In a carefully and insightfully designed study, *Iyengar et al.* [12] reported that doctors with high indegrees adopted early, and found evidence of social contagion primacy over social network ties even after controlling for targeted marketing efforts and time shocks. Adoption was affected by peers’ prescribing volume within the drug class, rather than by peers’ adoption per se. Due to credibility granted by experience with a newly introduced drug, doctors who prescribed a new drug more often were more reliable sources of information—not only did they follow their own recommendations, but they also had a large experimental base on which to found these recommendations.

*Lin et al.* [36] also found evidence for contagion. Overall, peers’ and opinion leaders’ adoption ratios were positively associated with the likelihood of early adoption. Of the three age-based groups (younger colleagues, compeers, and older colleagues), compeers’ and older colleagues’ adoption ratios were correlated with doctors’ likelihood of early adoption positively. Of the three tenure-based groups (junior colleagues, compeers, and senior colleagues), compeers’ and senior colleagues’ adoption ratios were associated with doctors’ likelihood of early adoption positively and significantly. The authors concluded that professional authority and informal interaction were equally important in the adoption process. While older doctors with longer tenures were granted professional authorities implicitly, on grounds of clinical experience, compeers in age and tenure exerted similar influences through more direct and frequent interactions. The authors argued that—besides senior colleagues, as almost exclusively advocated in the literature—peers should also be taken into account.

#### Practice characteristics

Eighteen of the 35 eligible studies investigate at least one practice-related characteristics of prescribers [1, 5–7, 11, 12, 14, 16–18, 20, 21, 24, 33, 35–37, 40].

*Location (urban or rural)* (n = 7) [1, 5, 6, 11, 16–18]. Rural practice locations may result in late new drug adoption—in contrast with their urban colleagues, rural doctors have fewer opportunities for professional interactions with peers, an important factor in the decision to initiate new treatments. The lower new drug utilisation rates in rural practices might also be explained by the lower number of visits by pharmaceutical sales representatives [5], due to geographic inaccessibility, or by the personal characteristics of doctors who elect to practice in rural communities.

At least partly, three of the seven eligible studies tended to favour the idea that urban practices adopt earlier and rural practices adopt later [1, 6, 18]. Tamblyn et al. [1] found that rural doctors were less likely to prescribe new drugs than their urban colleagues. Albeit only for two of six study drugs, Bourke and Roper [18] also reported an increase in time to adoption for practices in receipt of rural practice allowances. Finally, Groves et al. [6] concluded that the upper quartile of high-relative doctors may be best classified as doctors with urban practices. However, three other eligible studies found no evidence of early adoption of new drugs in urban areas [5, 16, 17], suggesting a reassuring efficiency of information dissemination. At the other extreme, two eligible studies found that rural practices adopted earlier [6, 11]. Greving et al. [11] reported that GPs who worked in rural areas were more likely to prescribe new medication than others, whereas Groves et al. [6] found that doctors classified as high-total new drug prescribers were more likely to operate in rural areas than elsewhere, possibly due to high patient—including elderly patient—loads.

*Type (solo or group/partnership)* (n = 7) [7, 11, 12, 20, 33, 35, 40]. In group/partnership practices, continuous professional stimulation and other social factors may accelerate the early adoption of new drugs. Joint responsibility for patients may promote the circulation of medical notes and allow for cross-fertilisation of therapeutic information [68], while daily personal contact with colleagues may provide an efficient channel for information transfer and evaluation. (For a discussion on the role of social networks in early adoption of new drugs, see the section ‘Contagion through social networks’.) However, the impact of group/partnership practices on new drug uptake is ambiguous in the literature [7, 11, 12, 20, 33, 35, 40].

On the one hand, Coleman et al. [7] reported that doctors who practised in groups/partnerships introduced new drugs on average 2.3 months earlier than doctors who practised solo—two other studies supported these findings [33, 40]. On the other, four of the seven eligible studies arrived at different conclusions. Iyengar et al. [12] and Kozyrskyj et al. [35] found no significant relationship between type of practice and early adoption of new drugs, whereas Dybdahl et al. [20] argued that the differences between solo and group/partnership practitioners disappeared after adjustment for practice size. Finally, Greving et al. [11] found that higher levels of new drug prescribing were associated with solo practices.

*Size* (n = 6) [1, 5, 16, 21, 36, 40]. Practice size may be measured in number of doctors, number of patients, prescribing volume, or number of services delivered. Intuitively, the larger the practice, the higher the probability of patients with conditions targeted by new drugs. However, only one of the six eligible studies favoured clearly the idea that larger practices adopt new drugs earlier than smaller practices. Steffensen et al. [40] found that the higher the number of patients, the higher the likelihood of early adoption. Tamblyn et al. [1] also found a modest but significant association between the two variables, but higher numbers of services delivered had different relationships for GPs and SPs, increasing the rate of new drug use for GPs, while reducing that for SPs. The other four studies found no empirical evidence for association between large practice size and early adoption of new drugs—whether size was measured by total prescribing volume [5, 21], by volume of prescribing within the therapeutic class of the new drug [21], by number of doctors [16, 36], or by number of patients [16, 21].

*Ownership (public or private), management (reformed or non-reformed), and orientation (for profit or not for profit)* (n = 4) [5, 24, 36, 37]. Ohlsson et al. [5] and Lin et al. [36] found that private practices were more likely to adopt new drugs earlier than public practices. In a similar setting, García et al. [24] reported that doctors in reformed institutions—under the management of distinct autonomous communities—adopted earlier than doctors in non-reformed institutions—operated by the National Institute of Health Management. This finding was very country specific, but supported the idea that autonomy may stimulate new drug diffusion. In contrast, Liu et al. [37] found that doctors in not-for-profit institutions were more likely to prescribe new drugs than doctors in for-profit institutions. The authors argued that for-profit hospitals delayed the introduction of new drugs in formularies—even if that hindered quality improvement—due to very strong incentives to reduce costs. However, this argument is at loggerheads with patients’ freedom to choose other hospitals, as well as in need of further evidence—the respective coefficients become insignificant, when standard errors are adjusted.

*Region* (n = 4) [12, 14, 24, 37]. Four studies examined regional impact on new drug diffusion through city or region dummies. Three of them found no differences across geographic locations [12, 14, 24], whereas Liu et al. [37] found that doctors in Taipei and central Taiwan were more likely to prescribe new drugs than doctors elsewhere in Taiwan. The authors argued that the speed of technological diffusion might depend on market size—most probably, pharmaceutical companies concentrated their marketing efforts on larger cities, with higher population densities and higher family incomes. However, considerable regional differences in Taiwan make generalisation difficult—regions may play an important role in more centralised countries and no role in more decentralised countries.

*Accreditation level* (n = 2) [12, 37]. Iyengar et al. [12] used a dummy variable to indicate whether doctors worked in—or were affiliated with—a university or teaching hospital, and found no significant association between level of institutional accreditation and new drug uptake. In contrast, Liu et al. [37] reported that accreditation levels—positively associated with practice size—had significant positive effects on the probability of prescribing new drugs. Consequently, academic medical centres had higher probabilities of prescribing new drugs than metropolitan and community hospitals. The authors argued that the higher the accreditation level, the higher the institution valued quality improvement—and the more inclined the institution was to prescribe new drugs. At the same time, higher levels of institutional accreditation—and, thus, higher market sizes—implied more significant marketing efforts from pharmaceutical companies.

*Diagnostic and therapeutic activities* (n = 2) [16, 40]. The two studies found that high volumes of diagnostic and therapeutic activities were associated positively with early adoption of new drugs [16, 40].

*Employee composition* (n = 2) [5, 18]. Ohlsson et al. [5] found that practices employing SPs as well as GPs were more likely to adopt new drugs early than practices employing GPs only—this finding supports the two-step model (see the section ‘Scientific orientation’). For two of six study drugs, Bourke and Roper [18] found small but significant lower adoption times for practices employing the assistance of a nurse or secretary—for the other four, no relationships were observed.

*Other* (n = 2) [1, 37]. Tamblyn et al. [1] found that higher *referral rates* were associated with significant reductions in SPs’ new drug prescribing rates. Most probably, SPs with higher referral rates treated patients with more comorbidities that required more careful and cautious prescribing. Liu et al. [37] reported that the higher the *healthcare market concentration*—that is, the higher the market share of certain practices in a given region—the higher the probability of prescribing new drugs. In contrast, the lower the healthcare market concentration—that is, the more competitive the market—the lower the probability of prescribing new drugs. According to Liu et al. [37], thus, high competition among healthcare providers—often advocated by policy makers—hindered new drug diffusion.

#### Drug characteristics

The majority of drug characteristics—improvement over existing therapies and the safety and efficacy of new drugs, for example—may require randomised controlled trials over long time periods and may not be measured easily. The five drug characteristics measurable quantitatively are the marketing budget of the pharmaceutical company assigned for the new drug, overall acceptance, therapeutic novelty, competition, and cost. Thirteen of the 35 eligible studies investigate at least one of the five quantitatively measurable drug charachteristics [1, 13, 15, 18, 20, 25, 27–30, 33, 37, 38].

*Marketing budget of the pharmaceutical company assigned for the new drug* (n = 7) [1, 13, 15, 28–30, 38]. The marketing budget of the pharmaceutical company assigned for a new drug affected early adoption in five of the seven studies, exerting significant and consistently signed influence—doctors were more likely to prescribe more heavily marketed drugs [13, 15, 28–30] ^i^. This influence was more pronounced for first entrants than for subsequent ones [15].

The other two studies arrived at different conclusions. Manchanda et al. [38] used monthly DTCA expenditure as proxy for aggregate marketing expenditure, and found no association with doctors’ adoption behaviour—not entirely surprising, since DTCA was zero in the first ten months after launch. Tamblyn et al. [1] also found no empirical evidence of relationships between advertising intensity—measured by detailing minutes and advertising pages—and early adoption of new drugs.

*Overall acceptance* (n = 6) [13, 18, 27, 29, 30, 38]. Six studies assessed the influence of time on new drug uptake by encompassing many potential influencers—for example, overall acceptance, growth in patient base, and the aggregate diffusion pattern in a geographic area. Manchanda et al. [38] and Liu and Gupta [13] controlled systematically for factors that might influence doctors’ adoption behaviour over time by adding time trend terms to their models—number of months lapsed since launch (for linear effects) and the same variable squared (for non-linear effects). The authors found unequivocally that temporal effects influenced new drug uptake significantly—however, the sign varied with the convexity of the temporal diffusion trend. Similarly, Glass [27] and Glass and Rosenthal [29] controlled for overall acceptance 12 months after launch by adding a market share variable. In line with intuition, the authors found that doctors were more likely to prescribe new drugs that were highly prescribed by other doctors. Bourke and Roper [18] also included an overall acceptance variable—the time-variant percentage of GPs who had adopted the study drug. The authors found a significant effect—for five of six study drugs, time to adoption increased with increases in the number of previous adopters. They explained the counterintuitive sign of the effect through market saturation—the longer the time, the fewer the GPs who had not already adopted. As an exception, Glass and Dalton [30] reported that market share 12 months after launch was not a significant variable in explaining the volume of new drug prescriptions.

*Therapeutic novelty* (n = 3) [25, 27, 33]. Garjón et al. [17] found that tiotropium—the only drug rated as therapeutic innovation—was adopted the most widely and quickest. Huskamp et al. [33] also reported that the four original antipsychotic formulations were quicker and more widespread adopted than reformulations. In contrast, Glass [27] showed that the prescribing level of the study drug six months after launch was not related to its therapeutic novelty. However, neither Huskamp et al. [33] nor Garjón et al. [25] could affirm that therapeutic novelty was the main reason for rapid new drug diffusion.

*Competition* (n = 1) [37]. Liu et al. [37] reported that the probability of prescribing new drugs was negatively associated with the level of competition in the pharmaceutical market—when new drugs entered therapeutic subgroups, the prescribing probability decreased by 0.2 percentage points. They argued that the profit margins of pharmaceutical companies increase with increases in the number of products they launch, given the unregulated character of the wholesale market—unlike the reimbursement price, the acquisition price is not determined by the regulator. Hence, profit margins will be larger for older drugs than for new drugs, and the probability that doctors will prescribe new drugs will be smaller.

*Cost* (n = 1) [20]. Dybdahl et al. [20] found that adoption time adjusted for number of listed patients was only weakly associated with the cost of new drug prescribing. The survey-based literature suggested that cost is less important than both safety and perceived efficacy [47], in general, and does not represent a significant barrier in the early adoption of new drugs [71]. Doctors try to balance efficacy and cost, but are not reluctant to prescribe higher cost, more effective drugs [44, 72]. Jacoby et al. [43] found that the most frequent early adopters of new drugs were the least cost conscious.

#### Patient characteristics

Patient characteristics such as age, gender, socioeconomic status, and the presence of comorbidities seem to influence new drug uptake. On the one hand, the empirical evidence is vast—on the other, characteristics of early receivers vary from drug to drug, with therapeutic goals and the target audience of the drug. An exhaustive review of the relevant literature is therefore impossible. Nine of the 35 eligible studies investigate at least one patient characteristics [1, 5, 11, 13, 14, 16, 32, 35, 37], their findings are discussed briefly in the following paragraphs.

*Age* (n = 9) [1, 5, 11, 13, 14, 16, 32, 35, 37]. Doctors’ likelihood of continuing to prescribe particular medications seemed to be influenced by patients’ age. Since elderly patients were more likely to experience side effects, doctors were less likely to prescribe new drugs to older patients [1, 16] and more likely to prescribe new drugs to younger patients [5, 11, 14]. Drugs generally designed for the elderly—to treat Alzheimer’s disease or arthritis, for example—were of course exceptions [32]. At the same time, three of the nine eligible studies found that patients’ age was not an important factor in new drug uptake [13, 35, 37].

*Gender* (n = 6) [5, 11, 14, 32, 35, 37]. Five of the six studies found that patient gender exerted small influence—if any—on doctors’ prescribing [11, 14, 32, 35, 37]—new drug characteristics and therapeutic goals were usually more important influencers. However, exceptions do occur—Ohlsson et al. [5] reported that more new drugs were prescribed to male than female patients.

*Health* (n = 4) [11, 14, 35, 37]. Patients’ health statuses—self-reported health, poor response to existing therapies, previous use of certain medications, and presence of comorbidities—evidently played an influential role in new drug uptake [11, 14, 37]. The variables reflecting significant associations included: patient referrals to cardiologists or internists, regardless of number of comorbidities [11]; higher comorbidity indexes; higher severities of diabetes, as measured by number of prescriptions per visit [37]; earlier onsets; and negative rather than positive symptoms in Mark et al.’s [14] multivariate model. However, chronic arthritic conditions made an exception, not playing an influential role in new drug prescribing for the majority of study drugs [35]. Generally, doctors seemed to consider individual contexts seriously, and patient convenience seemed to influence new drug uptake and promote earlier adoption among patients at desperate stages.

*Socioeconomic characteristics (income, education, and health insurance)* (n = 4) [5, 13, 14, 35]. By definition, socioeconomic statuses reflect patients’ economic and social positions relative to others, based on income, occupation, and education [73]. Three of the four eligible studies suggested that patients’ socioeconomic statuses influenced doctors’ prescribing behaviour irrespective of medical considerations [5, 13, 35]. High-income patients received new drugs earlier than others, not least because of their ability to pay for out-of-pocket treatments [5, 13, 35]. Privately insured patients or patients with a higher insurance index also seemed more likely to receive new drugs earlier than others [13]. However, Mark et al. [14] found no empirical evidence for positive impact of higher level of formal education and better insurance status on new drug uptake.

*Marital status* (n = 2) [5, 14]. Marital status may influence new drug uptake, but to lesser extent than socioeconomic status. Prescriptions for the treatment of high blood cholesterol were more probable among married or cohabiting patients [5], whereas prescriptions of atypical antipsychotics were equally likely among single patients and married or cohabiting ones [14].

*Race/ethnicity* (n = 2) [13, 14]. Association between race/ethnicity and socioeconomic status suggests association between race/ethnicity and new drug uptake. For example, non-African Americans were more likely to be treated with new medications than African Americans and Hispanics [13, 14].

## Discussion

For patients to receive the best possible care, doctors have to consider the risks and benefits of new drugs in conjunction with patient characteristics. Prescribing is a complex exercise, and early adoption of new drugs is the outcome of interactions among a wide variety of factors. The determinants of the decision to prescribe are interconnected in many—often conflicting—ways. This systematic review of the literature revealed a number of micro- and meso-level variables that produced consistent prediction of early adoption.

At *prescriber level*, the most significant influencers included interest in particular clinical or therapeutic areas, clinical trial participation, prescribing habits, targeted marketing efforts of pharmaceutical companies, and peer pressure through interpersonal communication.

Interest in particular clinical or therapeutic areas exerted influence on new drug uptake in the majority of cases. Early adoption of new drugs was more likely among SPs than GPs, whether drugs were special-purpose or for wide therapeutic spectra—new drugs diffused into general practice slower, because GPs preferred to follow the prescribing norms set by SPs. Partly related to clinical interest, clinical trial participation was also a powerful predictor of early adoption.

Prescribing habits were one of the most powerful predictors of new drug uptake. Not surprisingly, the greater the number of prescriptions written for all types of drugs or only within the therapeutic class of the new drug—and the wider the prescribing portfolio—the greater the chances of writing prescriptions for the new drug. High prescribing volumes in the therapeutic class of a new drug may lead to early adoption of that new drug for various reasons [13, 33]: (1) doctors are subject to enhanced adoption risk, because of large numbers of prescriptions; (2) doctors are likely to encounter patients who match the recommended patient profile of the new drug, because of diversified patient pools; (3) doctors may find it worthwhile, because the new drug may benefit other patients too; and (4) doctors are likely to be targeted by the marketing efforts of drug manufacturers. The prescribing volume of drugs by the pharmaceutical company introducing the new drug was also a powerful predictor of new drug uptake.

Pharmaceutical companies provide knowledge, increase product awareness, and direct further information acquisition. Accordingly, the size and efficiency of the marketing efforts targeted at doctors were very powerful predictors of new drug uptake. The reviewed studies showed almost unambiguously that pharmaceutical marketing had a significant and positive influence on prescribing. Among marketing efforts, detailing had the largest impact on new drug uptake, being robust to a wide variety of model specifications and datasets. Sampling, patient requests resulting from DTCA, and other direct-to-doctor marketing also influenced new drug uptake positively—however, their impact was rather modest. Promotional information continuing to influence early adoption—Manchanda and Honka [48] argued that pharmaceutical marketing involving billions of dollars is clearly here to stay.

Interpersonal communication—professional and social—also appeared to be a very important influencing factor, with information relayed through direct, personal contacts proving particularly powerful in new drug uptake. With the exception of Van den Bulte and Lilien [15], the other relevant eligible studies found empirical evidence of social contagion in new drug adoption effectuating in the form of consumption externality [7, 12, 13, 36, 38]. Interpersonal communication is possibly the richest medium of communication—and of influence over new drug uptake—and has important implications for both pharmaceutical companies and healthcare strategists. Pharmaceutical companies should continue to devote significant proportions of their marketing budgets to sales representatives, and should target customised and scientifically valuable information at key local opinion leaders. The practice of targeting heavy prescribers is a good strategy, common in the pharmaceutical industry—these prescribers not only have a high customer lifetime value, but also a high network value, through exerting more social contagion. At the same time, healthcare strategists should be very careful with projects that rely on electronic databases—efforts to utilise objective information to improve prescribing had ambiguous outcomes [47]. Preferably, healthcare strategists should rely on SPs to systematically disseminate new drug information and prescribing guidelines.

In addition, overseas qualifications and graduation from the youngest medical schools were also associated with higher rates of new drug uptake. More likely than not, unmeasured aspects of the training environment influenced new drug use—basic pharmacological training, policies related to drug detailing, relative financial contributions by pharmaceutical industries to training and research, or the educationally influential practices of attending doctors during formative training years [1].

There is a growing emphasis on evidence-based medicine suggesting that professional information counterbalances commercial information [7, 15]. In line with this shift, perceived scientific orientation, the number of professional journals read and attendance of specialist meetings and events also seemed to favour early adoption, with the exception of high-quality or low frequency PTAMs, which seemed to hinder it. However, drawing firm conclusions is difficult with such narrow research bases.

At *practice level*, only a few variables predicted early adoption consistently, in a small number of eligible studies—gaining insight into their roles would benefit from further research. The volume of diagnostic and therapeutic activities was consistently associated with new drug uptake. High volumes of diagnostic and therapeutic activities may be indicative of patient health severity, and of the need for early adoption of new drugs. Practices employing SPs as well as GPs—or employing more non-medical professionals—had higher rates of new drug uptake. Private healthcare institutions also seemed more likely to adopt new drugs earlier than public healthcare institutions.

At *drug level*, most characteristics may only be measured through randomised controlled trials over long periods of time. However, the marketing budgets pharmaceutical companies allocate for new drugs are a quantitatively measurable consistent predictor of new drug uptake. In line with expectations, the higher the marketing budgets, the faster the adoptions. Evidently, aggregate marketing expenditure correlates highly with the quality and quantity of marketing efforts targeted at specific doctors. Increasing the marketing budget of pharmaceutical companies may pay off, if marketing efforts are targeted efficiently at doctors—the marginal cost equals the marginal revenue of marketing.

Although research evidence was scant, overall acceptance and therapeutic novelty seemed to favour new drug uptake. One might reasonably assume that the factors affecting the uptake of highly innovative drugs might differ from the factors affecting the adoption of me-too drugs. In general, factors being consistently associated with new drug uptake were powerful predictors for both types of the drugs. Glass and Rosenthal [28] assessed the highly innovative drugs and me-too drugs in two distinct logistic regression models—their study was the only eligible one performing separate analysis in the function of innovation. The authors found that several variables were common to the adoption models for both types of drugs, including total prescribing volume, clinical trial participation, marketing budget of the pharmaceutical company assigned for the new drug, being a specialist in the respective specialty of the study drug and having office-based practices. The most striking difference between the two models was the role played by prescribing volume of the drugs by the same pharmaceutical company. This variable was not present in the model built for me-too drugs, yet it was the most important variable in the model assessing highly innovative drugs. The authors argued that this difference might indicate a degree of confidence and trust in that company, or in that company’s sales representatives, by the physician prescribing a therapeutically novel new drug from that company.

When highly innovative drugs are prescribed reducing safety and efficacy uncertainties is of crucial importance. Trust in the pharmaceutical company in providing a safe and efficacious novel drug seems to associate positively with new drug uptake [28]. Formal and informal contact with colleagues is another way to reduce uncertainty—empirical evidence suggests that these contacts are more important in higher risk drug adoption than in me-too drug uptake [50].

At *patient level*, consistent predictors of new drug uptake included young age and high socioeconomic status—high income, high level of formal education, and belonging to the majority race/ethnicity of the country. Furthermore, poor health status—either self-reported or due to comorbidity or unsatisfactory response to existing therapies—also promoted new drug uptake.

However, categorising early and late prescribers for a number of other variables was not possible, due to inconsistent results.

At *prescriber level*, age and gender were debated characteristics—in the majority of cases, no associations were found. Where associations were found, young age favoured early adoption, in line with intuition, and male doctors prescribed new drugs earlier than female doctors, most probably due to higher confidence with regard to the initiation of new medical treatments to achieve desired health outcomes [1, 74]. Professional age may correlate highly with age, but its impact on new drug uptake was even less obvious—half of the relevant studies reported positive associations, whereas the other half reported negative associations. At the same time, neither board certification nor hospital affiliation associated consistently with new drug uptake. With regard to the latter both formulary restrictions and the heightened professional orientation of hospital-affiliated doctors played a role in the adoption process, with formulary restrictions slightly more so, at least in Northern America.

Evidence on doctors’ positions, nationalities, current workplaces and the role CME plays in new drug diffusion was scant, and more research is needed to draw reliable conclusions. For example, with regard to nationalities, any future studies should control for regulatory environments—including pressure on doctors to prescribe generics cheaper than branded medicines—and country-level drug marketing expenditures.

At *practice level*, several variables yielded inconsistent results in quantifying the likelihood of new drug uptake. Group/partnership practices associated with new drug uptake in some studies, but not in all. In case of group/partnership practices, adjusting for practice size is essential in determining whether early adoption of new drugs stems from high number of patients or from continuous professional stimulation. Previous empirical research had rather suggested the former contention—group/partnership practices adopted new drugs early, if they adopted early at all, because they were (much more) likely to meet patients in need of new drugs [20]. The higher the number of patients a practice had, the higher the probability to consult patients who may have been candidates for new drugs—a conclusion other authors [7, 33, 40] may have drawn too, had they adjusted for practice size.

Practice location (rural or urban and central or peripheral) also did not predict consistently new drug uptake. Some studies indicated effective methods of information dissemination across geographical boundaries [5, 6] (for high-total new drug prescribers), [11, 12, 14, 16, 17, 24], while others not [1, 6] (for high-relative prescribers), [18, 37]. Modern communication technology, if used appropriately, most probably enables rural doctors to be as up-to-date as urban doctors—with abundant possibilities for CME and exchanges with colleagues, and with full access to information from pharmaceutical companies.

Practice size—measured by number of patients or prescribing volume—also did not associate consistently with new drug utilisation. This inconsistency is not only counterintuitive, but also at odds with the prescribing characteristics discussed earlier. Presumably, the innovative and conservative behaviours of individual doctors cancelled one another out, when summed up at practice level.

The impact of institutional accreditation on new drug diffusion was also controversial in the reviewed literature, one study finding positive association between level of institutional accreditation and new drug uptake, while another finding no empirical evidence for the association.

At *drug level*, the cost of new drug prescribing was only weakly associated with adoption time. Although competition in the pharmaceutical market was negatively associated with the probability of prescribing new drugs in one study, more research is needed to draw reliable conclusions.

At *patient level*, characteristics of early receivers varied from drug to drug, mostly with the therapeutic goal and target audience of the drug. Accordingly, neither patients’ gender nor their marital status produced consistent prediction.

This systematic review of the literature revealed a number of micro- and meso-level factors that produced either consistent or inconsistent prediction of early adoption. *Macro-level factors*, providing the framework for new drug diffusion, were so far not considered in this review. Many healthcare institutions and professional organizations, however, play a critical role in the diffusion process—their role is discussed briefly in the following paragraphs.

*Drug approval agencies* regulate and supervise pharmaceutical products, without their permission no drugs can be launched on the market. For example, in the EU a medicine can only be marketed if it has been approved either by the European Commission on the basis of the European Medicines Association’s (EMA) scientific opinion or by national competent authorities.

Once a regulatory agency has approved a new product, a pharmaceutical company will typically submit it for evaluation by *a payer* of some sort. Payers may be private insurance plans, government agencies such as the National Institute for Health and Care Excellence (NICE) in the UK, or health care organizations such as hospitals. Several public players apply cost-effectiveness analysis to guide reimbursement decisions [75]. Public payers have also started to negotiate risk-sharing agreements with pharmaceutical companies in an effort to mitigate the potential for an unexpectedly large budget impact due to incorrect assumptions and projections [75]. Reimbursement is, however, only one of the *supply-side controls* aimed at limiting the cost of reimbursed medicines to the authorities. Additional measures include price controls and limits on availability through the use of positive and negative lists.

Once the decision on the rate of reimbursement is made, *the demand for new drugs* still might be influenced by a number of ways, such as education, engineering, economic evaluation and enforcement [76]. Educational activities include, among others, development and distribution of prescribing guidance in which Drug and Therapeutic Committees (DTCs) play a crucial role. The DTCs may be regarded as a tool for promoting more efficient and rational use of medicines. Their drug recommendations are based on efficacy, safety, suitability and cost-effectiveness [77]. The recommendations of essential medicines, as a key component of rational use of medicines, should be enhanced by several complementary strategies, such as the ones defined in the Stockholm model [78].

With regard to demand-side measures, engineering activities include organizational or managerial interventions such as prescribing targets, whereas economic interventions include penalties on devolved budgets, financial incentives linked to prescribing targets, as well as differential patient co-payments [76]. Enforcement includes regulations by law such as mandatory generic substitution as well as prescribing restrictions [76] ^j^.

This systematic literature review has several possible *limitations*. First, it was undertaken by a single reviewer, heightening the potential for errors in the coverage and synthesis of the literature. Second, the chosen search strategies may have failed to identify prescription-based studies where new drug uptake was considered, but not as key focus. Third, prescription-based studies have advantages as well as disadvantages. They assess relationships based on very large data sets—however, data collected by health insurance funds are set up for insurance-related purposes. Consequently, the structure and content of the underlying data may not allow for answering certain research questions. Fourth, registry-based studies capture complex prescribing realities, but—without survey questionnaires and in-depth interviews—fail to encapsulate them comprehensively. Survey-based variables that complemented the registry-based variables were covered in this systematic literature review, but their role was peripheral, given possible self-reporting bias—missing independent validation, the quality of their evidence may have been suboptimal. Fifth, the studies reviewed here covered a range of drugs, prescribers, geographic regions, and nations—variance in results may have simply stemmed from differences in drugs, prescribers, or locations. In some cases, for example, the lack of concordance among study findings was evidently a straightforward consequence of the different drug characteristics and regulatory environments. Sixth, almost half of the eligible studies were US-based. The regulatory environment, the pharmaceutical companies’ determination, and doctors’ general attitude towards innovation evidently influence new drug diffusion. As yet, unfortunately, the literature offers no comparison of the influences of country-specific characteristics on new drug diffusion.

Most probably, doctors who coordinate patient care need to communicate regularly and effectively with many other doctors who share responsibility for patient care [79], allowing for the exertion of influence on new drug uptake. In future, the recent availability of administrative data of health insurance funds [79–81] might open new ways of identifying characteristics of early prescribers, by allowing researchers to construct and combine patient-sharing network data with doctors’ socio-demographic and professional characteristics on a large scale.

## Conclusions

This systematic literature review has provided insights into the factors that affect new drug uptake—primarily, doctors’ scientific orientation, prescribing habits, exposure to pharmaceutical marketing, and interpersonal communication. In relation to patient portfolios, doctors with younger patients, patients with higher socioeconomic statuses, and/or patients with poorer health statuses were more inclined to prescribe new drugs early. Some socio-demographic characteristics and practice-related factors also played an important role in the diffusion process. With so many different variables likely to influence new drug uptake, it is conceivable that each variable has only a moderate impact, which may explain the inconsistency of findings across studies.

Predicting doctors’ prescribing behaviour is an equally complex and multifactoral exercise. It continues to challenge researchers to gain clearer understanding of influencing factors and their interactions, to benefit all parties—whether patients, doctors, policymakers, or pharmaceutical companies. However, models with high numbers of variables and high explanatory power would be needed to help healthcare policy strategists to perfect interventions and pharmaceutical companies to optimise marketing budgets.

The fierce competition in the healthcare market is likely to stay—in search of profit, pharmaceutical companies are keen to add new products to their portfolios [3]. In contrast to Liu et al. [37], intuition suggests that the more competitive the markets, the more efforts pharmaceutical companies make to have their products prescribed by doctors. As a result, more new drugs would be prescribed in more competitive environments than in less competitive environments, particularly if the new drugs are innovative, cost-efficient and address unmet clinical needs in evolving markets. Moreover, early entry in combination with substantial market share would render late entry irrelevant—anecdotal evidence has it that doctors’ brains, already saturated by existing brands, can be penetrated neither by late entrants’ research and development activities nor by their marketing efforts. Thus, pharmaceutical companies may be best placed to develop both innovative drugs and models forecasting early adoption.

## Endnotes

^a^There are two studies [6, 35] in which both age and professional age are used. These studies, however, do not take interaction between age and professional age into account.

^b^However, Florentinus et al. [82] found that GPs were responsible for 20–30 per cent of first prescriptions.

^c^In the Netherlands, PTAMs bring together—on a regular and voluntary basis—pharmacists and GPs who work in the same catchment area. Their aim is to improve prescribing quality by deciding first-choice treatments [23].

^d^Time constraints and the broad range of conditions GPs treat do not allow them to review satisfactorily all relevant professional information—in itself time consuming and not always sufficiently convincing or technically clear. Although SPs rely on professional information more than GPs, they too make regular use of commercial information. For both SPs and GPs, sales representatives are often the first source of information, as well as an expedient means of keeping up-to-date and of acquiring and processing new drug-related information—even when doctors set to minimise the importance of sales representatives, to avoid distorted, selective, and overly positive information [47, 49]. Many interview-based studies— Prosser, Almond and Walley [49], Avorn, Chen, and Hartley [83], and M. Y. Peay and E. R. Peay [84], for example, all excluded from this review—confirmed the prominence of commercial information in early adoption of new drugs. Interactions with sales representatives had a particularly strong impact [8, 43, 49, 50, 72, 84]—early prescribers used sales representatives intensively [49, 72].

^e^García et al. [24] used drug expenditure as proxy for prescribing volume.

^f^These studies [55–61] were deemed ineligible because they did not meet all eligibility criteria. Azoulay [55], Berndt et al. [56], Cleary [57], and Lilien et al. [58] did not include any prescriber or practice characteristic; Gönül et al. [59] did not disclose anything about the nature of the drugs (new or old); the prescribing volume in Manchanda and Chintagunta [60] was indicative of old rather than new drugs; and Manchanda et al. [61] focused on the effectiveness of sales calls rather than the evaluation of factors affecting new drug uptake.

^g^These studies [62–67] were deemed ineligible because they did not meet the third eligibility criterion.

^h^Despite the unavailability of registry-based prescription data at the time, this study [7] was deemed eligible because it did meet the eligibility criteria—prescription data had been collected from pharmacies, rendering the analysis exempt from recall bias.

^i^Glass and Dalton [30] and Liu and Gupta [13] only had proxies for marketing spending. Glass and Dalton [30] included pharmaceutical company revenue as input variable, on the assumptions that larger companies had more marketing and sales resources for supporting clinical trials and selecting investigators, as well as for maintaining contact both during and after clinical trials. In a similar vein, Liu and Gupta [13] included journal advertising expenditure as input variable.

^j^Supply- and demand-size measures do not qualify for being quantitatively measurable predictors of new drug uptake—the reason why these measures were note reviewed in this article systematically.

## Author’s information

A two-year post-doctoral fellowship awarded by AXA Research Fund in 2011 is facilitating the author’s current project on networks of general practitioners and specialists, investigating the role of socio-demographic and network topological characteristics of doctors in professional interactions. This article is an integral part of this project—as well as of ongoing empirical research into characteristics of early prescribers of innovative oral anti-diabetic medication. The author’s enthusiasm for this area stems from an interest in the role social networks and key opinion leaders play, including in the pharmaceutical diffusion process. Initially, as a graduate in finance, the author worked with financial networks and systemic risk implications. This introduced her to the amazing potential of network theory, which she first exploited on large-scale financial networks and on social networks in telecommunications.

## Abbreviations

ATC: Anatomical therapeutic chemical classification system
CME: Continuing medical education
DTC: Drug and therapeutic committee
DTCA: Direct-to-consumer advertising
GP: General practitioner
PTAM: Pharmacotherapy audit meeting
SP: Specialist.
